# Influence of Socio-Economic Inequalities on Access to Renal Transplantation and Survival of Patients with End-Stage Renal Disease

**DOI:** 10.1371/journal.pone.0153431

**Published:** 2016-04-15

**Authors:** Wahida Kihal-Talantikite, Cécile Vigneau, Séverine Deguen, Muriel Siebert, Cécile Couchoud, Sahar Bayat

**Affiliations:** 1 EHESP School of Public Health, Sorbonne Paris Cité, Rennes, France; 2 CHU Pontchaillou, Service de néphrologie, Rennes, France; 3 Université de Rennes 1, UMR 6290, équipe Kyca, Rennes, France; 4 REIN Registry, Agence de la biomédecine, Saint Denis La Plaine, France; 5 EHESP School of Public Health, Sorbonne Paris Cité, EA MOS, Rennes, France; Clínica Universidad de Navarra, SPAIN

## Abstract

**Background:**

Public and scientific concerns about the social gradient of end-stage renal disease and access to renal replacement therapies are increasing. This study investigated the influence of social inequalities on the (i) access to renal transplant waiting list, (ii) access to renal transplantation and (iii) patients’ survival.

**Methods:**

All incident adult patients with end-stage renal disease who lived in Bretagne, a French region, and started dialysis during the 2004–2009 period were geocoded in census-blocks. To each census-block was assigned a level of neighborhood deprivation and a degree of urbanization. Cox proportional hazards models were used to identify factors associated with each study outcome.

**Results:**

Patients living in neighborhoods with low level of deprivation had more chance to be placed on the waiting list and less risk of death (HR = 1.40 95%CI: [1.1–1.7]; HR = 0.82 95%CI: [0.7–0.98]), but this association did not remain after adjustment for the patients’ clinical features. The likelihood of receiving renal transplantation after being waitlisted was not associated with neighborhood deprivation in univariate and multivariate analyses.

**Conclusions:**

In a mixed rural and urban French region, patients living in deprived or advantaged neighborhoods had the same chance to be placed on the waiting list and to undergo renal transplantation. They also showed the same mortality risk, when their clinical features were taken into account.

## Introduction

Renal transplantation is the optimal treatment for end-stage renal disease (ESRD). It is associated with increased quality of life [1,2], lower mortality and morbidity [3–5].

In developed countries, many studies found that age, gender and comorbidities [6,7] are associated with access to the renal transplant waiting list [8,9] and mortality [10]. Moreover, social inequalities in the access to the transplant waiting lists and to renal transplantation have been highlighted in the United States of America (USA) and United Kingdom (UK). These disparities may be related to several individual and neighborhood-related factors. Specifically, some works suggested a role for various non-medical individual factors, such as health insurance status [11], employment status [12] and education level [13]. Previous epidemiological studies also showed a social gradient of ESRD [14,15] and access to the transplant waiting list [16–19]. Indeed, people living in deprived neighborhoods are more likely to start renal replacement therapy and less likely to be wait-listed. However, the results of studies on access to renal transplantation after being waitlisted and on patients’ survival are contradictory [8,16]. The meta-analysis carried out by Morton et al. found consistent evidences that disadvantaged individuals with chronic kidney disease (CKD) have poorer access to quality treatment, including renal transplantation [20]. Studies in the USA suggest that the socio-economic status is a potential determinant of access to health care [21] that can influence the likelihood of placement on the renal transplant waiting list and of renal transplantation. In France, the access to diagnosis could be affected by neighborhood deprivation [22]; however, the national health insurance system covers the entire population and, therefore, ESRD treatment should not be limited by the patients’ socio-economic status. Moreover, medical and hospital costs for patients with CKD are completely covered (100%) and the reimbursement is regulated according to uniform rates, regardless of whether the patient is treated in public or private-sector nephrology facilities.

To our knowledge, few studies have investigated the impact of both patients’ medical features and non-medical contextual factors on the access to the renal transplant waiting list, renal transplantation and patients’ survival. These works assessed the influence of individual or neighborhood-related factors (deprivation, or degree of urbanization) on renal transplantation or on survival. However, most of them focused on racial disparities and investigated mainly the effect of poverty on these disparities [18,23].

In France, no study has assessed the social inequalities of access to renal transplantation. After a study on the socio-spatial inequalities of ESRD incidence in Bretagne [22], here we investigated the social inequalities in (i) the access to the renal transplant waiting list, (ii) access to renal transplantation after being wait-listed and (iii) patients’ survival, by taking into account both individual and neighborhood characteristics (neighborhood deprivation and degree of urbanization) of the smallest geographic unit (census block), in Bretagne, a French region.

## Material and Methods

### Study setting

This study was carried out in Bretagne, one of the administrative regions in France. Bretagne is a mixed urban and rural region, located in the western part of France, with a population of 3,094,000 inhabitants in 2006.

### Data source and participants’ selection

This study included a cohort of adult patients who lived in Bretagne and started dialysis (incident cases) between January 1, 2004 and December 31, 2009. This cohort was extracted from the French national “Réseau Epidémiologie et Information en Néphrologie” (REIN) registry [24,25]. The patients’ residential address was retrieved and matched to the corresponding census block. Preemptively transplanted patients were not included.

### Covariates

Five categories of variables were studied:

1. Demographic data: age group (18–39, 40–59, 60–69, ≥70 years), sex and occupational status.
2. Clinical features at first dialysis: body mass index (BMI), hemoglobin and serum albumin, primary renal disease (categorized in six groups: glomerulonephritis, pyelonephritis, diabetic nephropathy, hypertensive and vascular nephropathy, polycystic kidney disease and other causes/unknown) and comorbidities. The Comorbidities included in this analysis were: cardiovascular disease (coronary artery disease, peripheral vascular disease, congestive heart failure, arrhythmia, aortic aneurysm and cerebrovascular disease), diabetes, chronic respiratory disease, hepatic disease, active malignancy and physical disabilities (physical impairment of ambulation, para- or hemiplegia, blindness, member amputation).
3. Data concerning the medical follow-up in nephrology centers: ownership of nephrology facility where the first dialysis was performed (public university centers, public non-university centers and private centers), date of first dialysis, emergency *vs* planned first dialysis session, type of first dialysis (hemodialysis (HD) or peritoneal dialysis (PD)), date of placement on the waiting list, date of renal transplantation and date of death.
4. Blood type and Panel Reactive Antibody (PRA) for patients registered on the waiting list and donor type (deceased or living) for patients who underwent renal transplantation.
5. Two neighborhood characteristics at the census-block level: (i) degree of urbanization (rural/ urban typology) and (ii) socio-economic deprivation index. The residential census block of each patient was classified as urban or rural [22] using an approach inspired from the study by Van Eupene al., 2012 [26]. The urban/rural typology was defined by combining two criteria of rural/urban classification: the population density, using the OECD typology (Organization for Economic Co-operation and Development) [27] and land cover [28] (for more details, see Kihal et al., 2015 [22]). For the socio-economic deprivation index, socio-economic and demographic data were obtained from the 2006 census at the census-block level. To characterize the neighborhood deprivation level, a deprivation index was used that included variables related to education, income, occupation, unemployment and immigration to cover and capture the different dimensions of deprivation. Successive principal-component analyses were performed to calculate the deprivation index, based on Lalloue et al. [29], in each considered geographic area (rural/urban). The measure of neighborhood deprivation was categorized in three groups (low, moderate or high deprivation) according to the tertiles of the index distribution [22].

### Outcomes measures

The outcomes of interest included:

1. Access to the renal transplant waiting list: patients placed on the waiting list before starting dialysis were considered to be wait-listed at first dialysis. Time to wait-listing was calculated from the date of first dialysis. Not wait-listed patients were censored at the date of death or at the end of the follow-up period (December 31, 2011).
2. Access to renal transplantation after placement on the list: patients who received renal transplant from a living donor were excluded from the analyses. Time to renal transplantation was calculated from the date of placement on the waiting list. Non-transplanted patients were censored at the date of death or at the end of the follow-up period.
3. Survival: time to death was calculated from the date of the first dialysis. Living patients were censored at the end of the follow-up period. Renal transplantation was considered as a time- dependent covariate.

### Statistical analyses

Cox proportional hazards models were used to identify the factors associated with the likelihood of (i) being placed on the waiting list, (ii) being transplanted after placement on the waiting list and (iii) survival. Patients with missing data were excluded from the analyses.

First, univariate Cox regression analyses were performed to assess associations between the outcomes and the patients’ characteristics (including neighborhood data). Then, multivariate Cox models were constructed using variables with a p-value lower than 0.2 in univariate analyses and variables that were selected *a priori*, based on literature findings (gender and neighborhood deprivation). Results were reported as hazard ratios (HR) with 95% confidence interval (CI) and p-values. Statistical significance was identified by a p-value lower than 0.05. All analyses were performed using the STATA software (version11.2).

### Ethics statement

This retrospective study was approved by the French Biomedecine Agency and included patients’ data that were anonymized and de-identified directly in the REIN database before extraction for the analysis.

Patients and the associated data were extracted from the French REIN registry that was approved by CNIL (Commission Nationale de l’Information et des Libertés) in 2010. REIN is registered with the CNIL under the following number: 903188 Version 3.

## Results

### Participants’ characteristics

Data concerning 2006 incident patients who lived in Bretagne and started dialysis between January 2004 and December 2009 were extracted from the REIN registry. By the end of 2011, 27% of them were registered on the transplant waiting list and 24% had received a kidney transplant mostly from deceased donors (five living donors). Moreover, 931 patients (46%) died during the follow-up period.

Among the 2006 patients, 56.1% were older than 70 years (mean age: 67.3 years), 60.6% males, 79.1% without occupation, 26% had diabetes, 54.5% had cardiovascular diseases, 47.2% lived in a rural area, 50% in census-blocks with high socio-economic deprivation and 18.7% in areas with low deprivation (Table 1).

**Table 1:** Patients’ characteristics at first dialysis and their association with access to the waiting list (univariate and multivariate Cox analyses).

Patients’ characteristics	Number (%)	Univariate analysis		Multivariate analysis	
	2006	HR (95%CI)	p-value	HR (95%CI)	p-value
**Age**					
18–39	122 (6.1)	54.52 [36.7–81.1]	<0.001	29.81 [18.1–49.1]	<0.001
40–59	425 (21.2)	38.10 [24.4–54.9]	<0.001	23.50 [15.2–36.4]	<0.001
60–69	334 (16.6)	12.15 [8.2–18.0]	<0.001	11.00 [7.01–17.2]	<0.001
≥70	1125 (56.1)	1		1	
**Sex **					
Men	1216 (60.6)	1.06 [0.9–1.2]	0.49	1.01 [0.8–1.2]	0.9
Women	790 (39.4)	1		1	
**Occupational status**					
Yes	264 (14.3)	8.38[7.0–10.2]	<0.001	1.61 [1.3–2.0]	<0.001
No	1587 (85.7)	1		1	
**BMI † (kg/m2)**					
<20	262 (14)	0.98 [0.7–1.3]	0.87	-	
[20–25[	783 (41.8)	1		-	
[25–30 [	537 (28.7)	0.85 [0.7–1.1]	0.14	-	
[30–35 [	212 (11.3)	1.02 [0.8–1.3]	0.85	-	
≥35	79 (4.2)	0.46 [0.3–0.8]	0.008	-	
**Serum albumin (g/dl)**					
<30	347 (20.6)	0.47 [0.3–0.6]	<0.001	-	
≥30	1334 (79.4)	1			
**Hemoglobin (g/dl)**					
<10	600 (32.7)	1.04 [0.8–1.3]	0.64	-	
[10–12[	879 (47.9)	1		-	
≥12	356 (19.4)	1.31 [1.1–1.6]	0.02	-	
**Type of dialysis**					
HD	1756 (87.5)	0.85 [0.7–1.1]	0.20	-	
DP	250 (12.5)	1		-	
**Emergency first dialysis session**					
Yes	558 (71.4)	0.67 [0.5–0.8]	<0.001	0.59 [0.5–0.8]	<0.001
No	1393(28.6)	1		1	
**Ownership of dialysis facility**					
Private	791(39.4)	2.16 [1.8–2.6]	<0.001	1.4 1[1.1–1.7]	0.001
Public, non-university	943(47.0)	1		1	
Public, university	272 (13.6)	0.52 [0.4–0.8]	0.001	0.64 [0.4–1.0]	0.06
**Primary renal disease**					
APKD ^**‡**^	76 (3.8)	1		1	
Hypertensive & vascular	481 (24.0)	0.14 [0.1–0.2]	<0.001	0.72 [0.5–1.0]	0.07
Other & unknown	786 (39.2)	0.26 [0.2–0.3]	<0.001	0.62 [0.5–0.8]	<0.001
Diabetes	187 (9.3)	0.25 [0.2–0.4]	<0.001	0.67 [0.4–0.98]	0.04
Glomerulonephritis	267 (13.3)	0.64 [0.5–0.8]	0.001	0.85 [0.6–1.1]	0.28
Pyelonephritis	108 (5.4)	0.59 [0.4–0.8]	0.003	0.77 [0.5–1.1]	0.19
**Cardiovascular disease**					
Yes	1058 (54.5)	0.20 [0.2–0.3]	0.001	0.52 [0.4–0.7]	<0.001
No	883 (45.5)	1		1	
**Chronic respiratory disease**					
Yes	239 (87.5)	0.38 [0.3–0.6]	<0.001	-	
No	1668(12.5)	1		-	
**Active malignancy**					
Yes	217 (11.4)	0.18 [0.1–0.3]	<0.001	0.28 [0.2–0.5]	<0.001
No	1689 (88.6	1		1	
**Diabetes**					
Yes	505 (26.0)	0.49 [0.4–0.6]	<0.001	-	
No	1437 (74.0)	1		-	
**Physical disabilities**					
Yes	335 (17.6)	0.18 [0.1–0.3]	<0.001	0.48 [0.3–0.8]	0.002
No	1566 (82.4)	1		1	
**Hepatic disease**					
Yes	1 832 (96.3)	0.21 [0.1–0.5]	0.001	0.23 [0.1–0.6]	<0.001
No	70 (3.7)	1		1	
**Neighborhood deprivation**					
Low	75(18.7)	1.40 [1.1–1.7]	0.002	1.04 [0.8–1.3]	>0.5
Moderate	628(31.3)	1.06 [0.9–1.3]	0.53	1.14 [0.9–1.4]	0.7
High	1003(50.0)	1		1	
**Degree of urbanization**					
Rural	946 (47.2)	1		-	
Urban	1060 (52.8)	0.86 [0.7–1.0]	0.09	-	

^†^ Body Mass Index

‡ polycystic kidney disease

HD: hemodialysis; PD: peritoneal dialysis

Among the patients placed on the waiting list (n = 546), 5.9% were older than 70 years, 61.5% were males and 56.1% without occupation, 16.3% had diabetes, 21.6% had cardiovascular diseases, 50.7% lived in a rural area, 46.1% were from highly disadvantaged and 23.6% from less disadvantaged census-blocks.

### Access to the renal transplant waiting list (Table 1)

#### (a) Univariate analysis

Occupation, young age, high hemoglobin level (≥12 g/dl) and private ownership of the nephrology facility were significantly associated with higher access to the transplant waiting list. The presence of co-morbidities, physical disabilities, low serum albumin level (<30 g/dl), high BMI value (≥35kg/m²), emergency first dialysis (*vs* planned one) and all primary renal diseases (compared with polycystic kidney disease) were significantly associated with a lower probability of being wait-listed. Patients living in less disadvantaged census-blocks had higher access to the waiting list than those living in highly disadvantaged areas. Similarly, access to the waiting list was higher for patients living in urban areas than for those living in rural census-blocks.

#### (b) Multivariate analysis

Young patients (18–39 years) were more likely to be placed on the waiting list (aHR = 29.81 95%CI: [18.1–49.1]). Patients with a cardiovascular disease, hepatic disease, active malignancy or physical disability were less likely to be placed on the list than patients without comorbidity (adjusted HR, aHR = 0.52 95%CI: [0.4–0.7]; aHR = 0.23 95%CI: [0.1–0.6]; aHR = 0.28 95%CI: [0.2–0.5]; aHR = 0.48 95%CI: [0.3–0.8], respectively). Similarly, emergency first dialysis session was still associated with a lower probability of being wait-listed (aHR = 0.59 95%CI: [0.5–0.8]). Patients followed in private facilities were 41% more likely to be registered on the waiting list than patients followed in public non university centers. Conversely, neighborhood deprivation was not significantly associated with access to the waiting list in multivariate analysis.

### Access to deceased donor renal transplantation after being wait-listed (Table 2)

#### (a) Univariate analysis

Patients aged ≥70 years or with the A or AB blood groups (compared with the O group) had higher access to renal transplantation. Conversely, the presence of diabetes and active malignancy at first dialysis, diabetic nephropathy (compared with polycystic kidney disease) and hyperimmunisation (PRA ≥85%) were associated with lower access to renal transplantation. Neighborhood deprivation and rural/urban typology were not significantly associated with access to renal transplantation.

**Table 2:** Association between patients’ characteristics and access to renal transplantation after placement on the waiting list (univariate and multivariate Cox analyses).

Patients’ characteristics	Number (%)	Univariate analysis		Multivariate analysis	
	546	HR (95%CI)	p-value	HR (95%CI)	p-value
**Age**					
18–39	104 (19.0)	1		1	
40–59	302 (55.3)	1.00 [0.8–1.3]	0.93	0.86 [0.7–1.1]	0.16
60–69	108 (19.8)	1.04[0.8–1.4]	0.74	0.89 [0.7–1.2]	0.20
≥70	32 (5.9)	1.61[1.1–2.4]	0.02	1.72 [1.1–2.6]	0.01
**Gender**					
Men	336 (61.5)	1.01 [0.8–1.2]	0.90	0.95 [0.8–1.2]	0.71
Women	210 (38.5)	1		1	
**Occupation status**					
No	281 (56.1)	1			
Yes	220 (43.9)	1.12 [0.9–1.3]	0.23		
**Blood group**					
O	258 (47.3)	1		1	
A	207 (37.9)	1.95 [1.6–2.4]	<0.001	2.17 [1.8–2.8]	<0.001
B	22 (4.0)	0.69 [0.5-.0.9]	0.02	0.70 [0.5–0.9]	0.045
AB	59 (10.8)	2.08 [1.3–3.3]	0.002	1.89 [1.1–3.13]	0.047
**Panel Reactive Antibody (PRA)**					
%<85	525 (96.1)	1		1	
≥85	21 (3.9)	0.63 [0.4–1.0]	0.05	0.57 [0.4–0.93]	0.027
BMI*(kg/m2)					
<20	73 (13.9)	0.83 [0.6–1.1]	0.2	-	
[20–25[	232 (44.1)	1		-	
[25–30 [	141 (26.8)	0.94 [0.7–1.2]	0.61	-	
[30–35 [	67(12.7)	0.78 [0.6–1.1]	0.11	-	
≥35	13 (2.5)	0.93 [0.5–1.7]	0.81	-	
**Serum albumin (g/dl)**					
<30	52 (11.0)	0.76 [0.5–1.1]	0.09	-	
≥30	420 (89)	1		-	
**Hemoglobin (g/dl)**					
<10	163 (31.8)	1.01 [0.8–1.3]	0.85		
[10–12[	232 (45.2)	1			
≥12	118 (23)	0.93 [0.7–1.2]	0.58		
**Type of dialysis **					
HD	473 (86.6)	0.88 [0.7–1.2]	0.37		
DP	73 (13.4)	1			
**Ownership of dialysis facility**					
Private	318 (58.2)	1.17 [1.0–1.4]	0.09	-	
Public, non-university	198 (36.3)	1		-	
Public, university	30 (5.5)	1.33 [0.9–2.1]	0.19	-	
**Emergency first dialysis session**					
Yes	115 (21.1)	0.95 [0.8–1.2]	0.67		
No	423 (77.5)	1			
**Primary renal disease**					
APKD**	110 (20.2)	1		-	
Hypertensive & vascular	61 (11.2)	1.03 [0.7–1.4]	0.83	-	
Other &unknown	164 (30.0)	0.71 [0.6–0.99]	0.04	-	
Diabetes	41 (7.5)	0.65 [0.4–0.98]	0.04	-	
Glomerulonephritis	124 (22.7)	0.91 [0.7–1.2]	0.51	-	
Pyelonephritis	46 (8.4)	0.98 [0.7–1.4]	0.92	-	
**Cardiovascular disease **					
Yes	114 (21.6)	0.84 [0.7–1.1]	0.15	0.74 [0.6–0.9]	0.017
No	414 (78.4)	1		1	
**Chronic respiratory disease**					
Yes	28 (5.3)	0.67 [0.43–1.0]	0.07	-	
No	495(94.6)	1		-	
**Active malignancy **					
Yes	13 (2.5)	0.46 [0.2–0.97]	0.04	-	
No	504 (97.5)	1		-	
**Diabetes**					
Yes	86 (16.3)	0.70 [0.5–0.9]	0.01	-	
No	443 (83.7)	1			
**Physical disabilities**					
Yes	19 (3.6)	0.58 [0.3–1.0]	0.07	-	
No	506 (96.4)	1		-	
**Hepatic disease**					
Yes	5 (1.0)	0.72 [0.2–2.3]	0.57		
No	512 (99.0)	1			
**Neighborhood deprivation**					
Low	129 (23.6)	0.88 [0.7–1.1]	0.29	0.86 [0.7–1.1]	0.26
Moderate	165 (30.2)	0.99 [0.8–1.2]	0.95	1.03 [0.8–1.3]	0.90
High	252 (46.2)	1		1	
**Degree of urbanization**					
Rural	277(50.7)	0.97 [0.8–1.2]	0.76		
Urban	269 (49.3)	1			

* Body Mass Index

** Polycystic kidney disease

HD: hemodialysis; PD: peritoneal dialysis

#### (b) Multivariate analysis

Patients aged ≥70 years (compared with the 18–39 year/old group) were 72% more likely to receive a transplant. Conversely, all clinical data at first dialysis, type of primary renal disease and type of first dialysis were no longer associated with the probability of undergoing transplantation. Among comorbidities, only cardiovascular diseases remained significantly associated with a lower probability of transplantation (aHR = 0.74 95%CI: [0.6–0.9]).

Patients with A or AB blood group were still more likely (two times) to receive a kidney transplant than O group patients. Conversely, B blood group and high degree of immunization were associated with a lower probability of receiving a renal transplant (aHR = 0.70 95%CI: [0.5–0.9]; aHR = 0.57 95%CI: [0.4–0.93], respectively).

After adjustment for the patients’ features, the likelihood of renal transplantation was not significantly different in the different socio-economic areas, although it was slightly lower for patients living in advantaged neighborhoods than for patients living in deprived areas (aHR = 0.86 95%CI: [0.7–1.1]).

### Patients’ survival (Table 3)

#### (a) Univariate analysis

Low BMI (<20 kg/m²), low serum albumin level (<30 g/dl), presence of comorbidities, physical disabilities, emergency first dialysis and all primary renal diseases (compared with polycystic kidney disease) were significantly associated with higher mortality risk. Compared with patients followed in public non university centers, the mortality risk was lower for patients followed in private centers and higher for those followed in public university facilities. Transplantation during the follow-up period was associated with lower mortality risk. Neighborhood deprivation was significantly associated with higher mortality; conversely, the degree of urbanization was not associated with the mortality risk.

**Table 3:** Association between patients’ characteristics at first dialysis and mortality (univariate and multivariate Cox analyses).

Patients’ characteristics	Univariate analysis		Multivariate analysis	
	HR (95%CI)	p-value	HR (95%CI)	p-value
**Age**				
18–39	0.05 [0.0–0.1]	<0.001	0.18 [0.1–0.4]	<0.001
40–59	0.18 [0.1–0.2]	<0.001	0.40 [0.3–0.5]	<0.001
60–69	0.42 [0.3–0.5]	<0.001	0.47 [0.4–0.6]	<0.001
> = 70	1		1	
**Sex**				
Men	1.11 [0.98–1.3]	0.09	1.1 [0.9–1.34]	0.120
women	1		1	
**Occupation status**				
Yes	0.09 [0.06–0.15]	<0.001	-	-
No	1			
BMI*(kg/m2)				
<20	1.27 [1.04–1.6]	0.01	1.43 [1.-1.8]	0.003
[20–25[	1		1	
[25–30 [	1.00 [0.8–1.2]	0.92	0.90 [0.7–1.1]	0.30
[30–35 [	0.99 [0.8–1.2]	0.96	0.87 [0.7–1.1]	0.33
≥35	0.82 [0.6–1.0]	0.33	0.63 [0.4–1.04]	0.06
**Serum albumin (g/dl)**				
<30	1.80 [1.5–2.1]	<0.001	1.3 [1.1–1.6]	0.003
≥30	1		1	
**Hemoglobin (g/dl)**				
<10	1.05 [0.9–1.2]	0.48	-	
[10–12[	1		-	
≥12	0.83 [0.7–1.0]	0.06	-	
**Type of dialysis **				
HD	0.82 [0.7–0.99]	0.05	0.77 [0.6–0.96]	0.026
DP	1		1	
**Emergency first dialysis session**				
Yes	1.31 [1.1–1.5]	<0.001	-	-
No	1		-	
**Renal transplantation**				
Yes	0.08 [0.05–0.1]	<0.001	0.25 [0.2–0.4]	<0.001
No	1		1	
**Primary renal disease**				
APKD**	1		1	
Hypertensive & vascular	5.38 [3.6–8.0]	<0.001	2.50 [1.5–4.17]	<0.001
Other & unknown	4.44 [3.0–6.6]	<0.001	2.70 [1.6–4.5]	<0.001
Diabetes	4.32 [2.8–6.6]	<0.001	2.64 [1.5–4.6]	<0.001
Glomerulonephritis	2.16 [1.4–3.3]	<0.001	1.72 [0.99–2.99]	0.053
Pyelonephritis	2.45 [1.5–4.0]	<0.001	2.32 [1.3–4.28]	0.007
**Ownership of dialysis facility**				
Private	0.64 [0.6–0.7]	<0.001	-	-
Public, non-university	1		-	-
Public university	1.49 [1.2–1.8]	<0.001	-	-
**Cardiovascular disease**				
Yes	3.40 [2.9–3.9]	<0.001	1.83 [1.5–2.2]	<0.001
No	1		1	
**Chronic respiratory disease**				
Yes	1.76 [1.5–2.1]	<0.001	1.40 [1.1–1.7]	0.002
No	1		1	
**Active malignancy**				
Yes	2.33[1.9–2.8]	<0.001	1.51 [1.2–1.89]	<0.001
No	1		1	
**Diabetes**				
Yes	1.44[1.2–1.7]	<0.001	-	
No	1		-	
**Physical disabilities**				
Yes	2.94 [2.5–3.4]	<0.001	1.8 [1.5–2.2]	<0.001
No	1		1	
**Hepatic disease**				
Yes	1.64 [1.2–2.2]	0.001	-	
No	1		-	
**Neighborhood deprivation**				
Low	0.82 [0.7–0.98]	0.03	0.98 [0.8–1.24]	0.9
Moderate	1.02[0.9–1.2]	0.73	1.13 [0.95–1.35]	0.13
High	1		1	
**Degree of urbanization**				
Urban	1.06[0.9–1.2]	0.35		
Rural	1			

* Body Mass Index

** Polycystic kidney disease

HD: hemodialysis; PD: peritoneal dialysis

#### (b) Multivariate analysis

Patients aged between 18 and 39 (compared with the ≥70 year/old group) had a lower risk of death (aHR: 0.18[0.1–0.4]). Patients with a cardiovascular disease, respiratory disease, active malignancy, or physical disabilities had a higher mortality risk (aHR = 1.83 95%CI: [1.5–2.2]; aHR = 1.40 95%CI: [1.1–1.7]; aHR = 1.51 95%CI: [1.2–1.89]; aHR = 1.81 95%CI: [1.5–2.2], respectively). Low BMI values (<20 g/dl) were associated with higher probability of death (aHR = 1.43 95%CI: [1.0–1.8]). Transplantation during the follow-up period was associated with lower probability of death (aHR = 0.25 95%CI: [0.2–0.4]).

After adjustment for the patients’ clinical features, the likelihood of mortality was no longer associated with neighborhood deprivation, although it was slightly lower among patients living in advantaged than among those living in deprived areas (aHR = 0.98 95%CI: [0.8–1.2]).

## Discussion

This first contextual study in France on the role of socio-economic factors in the access to renal transplantation or patients’ survival shows that, after taking into account the patients’ clinical features, neighborhood deprivation and degree of urbanization are not associated with access to the renal transplant waiting list, transplantation after placement on the list or survival.

To our knowledge, no other study on the access to renal transplantation has taken into account both patients’ clinical features and neighborhood characteristics (socio-economic level and urbanization degree) at a fine spatial level in mixed urban and rural areas. Indeed, most studies focused either on racial disparities or on poverty [18,23], without considering the urbanization degree [8,16,30,31]. Moreover, in contrast with previous reports from the USA, the UK and Scotland [8,19,32–37], our study took into account all major comorbidities.

Although our univariate analysis showed that patients living in highly deprived neighborhoods had less chance to be placed on the waiting list, this association did not remain after taking into account other patients’ characteristics. In the USA [16–18,38] and in the UK [8,19], all studies found that patients living in a highly deprived neighborhood were less likely to be placed on the waiting list. Moreover, a higher social adaptability index (SAI) was associated with increased likelihood of being wait-listed [16].

Our findings show that the likelihood of renal transplantation after placement on the list and risk of death were slightly, but not significantly, lower among patients living in advantaged neighborhoods. Contradictory results were reported by previous epidemiological studies. Indeed, while some authors found that patients had an equal chance of transplantation, regardless of their socioeconomic status [8,39], others showed that patients living in advantaged neighborhoods had a greater likelihood of receiving a transplant [16,31]). Conversely, neighborhood poverty [40] or lower median income [17] was associated with a reduced probability of transplantation [17]). Another study found that neighborhoods with low level of deprivation were associated with reduced mortality risk [30] and that mortality was higher for patients living in the poorest areas [40] only among Asians and Pacific Islanders. On the other hand, another work reported that the risk of death was lower among patients living in deprived neighborhoods [31].

Overall, studies carried out in the USA and UK show that neighborhood deprivation plays an important role; conversely, our study suggests that neighborhood deprivation is not a determinant factor for receiving renal transplantation. These conflicting results may be due to health care system differences between the USA, the UK and France (universal health care system). Indeed, this finding is plausible because in France, the national health insurance system covers the entire population. Moreover, people living in deprived neighborhoods have often more comorbidities (diabetes, cardiovascular diseases…) and malnutrition. These factors can limit the access to the waiting list. Therefore, if the patient’s medical condition is not taken into account during the analysis, the neighborhood deprivation effect may in reality reflect the comorbidity influence [14]. In addition, neighborhood deprivation may be associated with nephrologists’ clinical practice patterns. Our study was performed in Bretagne where there are two transplantation centers. This might have reduced the bias related to clinical practice variations and increased the possibility to analyze factors directly related to patients.

These conflicting results could also be partially explained by the use of different deprivation measures (composite indexes [31], SAI [16,30], Carstairs score [8] or Townsend [19], poverty level [18,38,40] and income level [17]) to study how the access to the waiting list and to kidney transplant is affected by socio-economic variables. In our work, we used a neighborhood deprivation index that included variables related to education, income, occupation, unemployment and immigration to cover different dimensions of deprivation in rural and urban settings. This deprivation index has been validated and previously used to demonstrate socio-economic gradients in the incidence of ESRD in Bretagne [22] and of infant mortality in Lyon [41,42].

The second main finding of our study is that the degree of urbanization, like the neighborhood socio-economic features, does not influence the access to the transplant waiting list, to transplantation after being wait-listed and patients’ survival. A few recent studies revealed conflicting results. A study found that the urbanization degree of the patient’s residence was not associated with the time on the waiting listing [38]. Conversely, other works have shown that the likelihood of placement on the list [43] and of transplantation [43] was slightly, but significantly higher for people living in rural areas than for those residing in urban areas. Studies in the USA found that white non-Hispanic and Native American patients living in rural areas were more likely to undergo transplantation than those living in urban areas [44]. Conversely, in Rotterdam, low urbanization grade significantly and negatively influenced the chance of living donor transplantation [39].

Our study has some limitations. Race/ethnic differences were not recorded in the French ESRD registry because the French legislation does not allow collecting this kind of information. Data about the individual socio-economic status and about individual preferences were not available and were thus not included in our analysis. However, we chose a fine geographical scale, designed to be as homogeneous as possible in terms of socio-economic characteristics. The census block homogeneity allowed minimizing the ecological bias and the results can be considered as close as possible to what can be observed at the individual level.

## Conclusion

In this study, we assessed social inequalities at a fine scale in a mixed rural and urban French region. Our results show that, after taking into account all major patients’ clinical characteristics, patients living in deprived neighborhoods and those living in advantaged ones had the same chance to be placed on the waiting list, to be transplanted and the same mortality risk. In France where everybody is covered by the national health insurance, the association observed, in univariate analysis, between higher neighborhood deprivation and lower access to renal transplantation is more related to the patients’ clinical features than to socio-economic factors, or nephrologists’ clinical practices.

## References

[1] Evans JM, Newton RW, Ruta DA, MacDonald TM, Morris AD. Socio-economic status, obesity and prevalence of Type 1 and Type 2 diabetes mellitus. Diabet Med. 2000;17: 478–480. 10975218

[2] Laupacis A, Keown P, Pus N, Krueger H, Ferguson B, Wong C, et al A study of the quality of life and cost-utility of renal transplantation. Kidney Int. 1996;50: 235–242. 8807593 10.1038/ki.1996.307

[3] Port FK, Wolfe RA, Mauger EA, Berling DP, Jiang K. Comparison of survival probabilities for dialysis patients vs cadaveric renal transplant recipients. JAMA. 1993;270: 1339–1343. 8360969

[4] Wolfe RA, Ashby VB, Milford EL, Ojo AO, Ettenger RE, Agodoa LY, et al Comparison of mortality in all patients on dialysis, patients on dialysis awaiting transplantation, and recipients of a first cadaveric transplant. N Engl J Med. 1999;341: 1725–1730. 10.1056/NEJM199912023412303 10580071

[5] Meier-Kriesche H-U, Schold JD, Srinivas TR, Reed A, Kaplan B. Kidney transplantation halts cardiovascular disease progression in patients with end-stage renal disease. Am J Transplant. 2004;4: 1662–1668. 10.1111/j.1600-6143.2004.00573.x 15367222

[6] Couchoud C, Bayat S, Villar E, Jacquelinet C, Ecochard R, REIN registry. A new approach for measuring gender disparity in access to renal transplantation waiting lists. Transplantation. 2012;94: 513–519. 10.1097/TP.0b013e31825d156a 22895611

[7] Bayat S, Macher MA, Couchoud C, Bayer F, Lassalle M, Villar E, et al Individual and regional factors of access to the renal transplant waiting list in france in a cohort of dialyzed patients. Am J Transplant. 2015;15: 1050–1060. 10.1111/ajt.13095 25758788

[8] Oniscu GC, Schalkwijk AAH, Johnson RJ, Brown H, Forsythe JLR. Equity of access to renal transplant waiting list and renal transplantation in Scotland: cohort study. BMJ. 2003;327: 1261 10.1136/bmj.327.7426.1261 14644969 PMC286245

[9] Garg PP, Furth SL, Fivush BA, Powe NR. Impact of gender on access to the renal transplant waiting list for pediatric and adult patients. J Am Soc Nephrol. 2000;11: 958–964. 10770976 10.1681/ASN.V115958

[10] Miskulin DC, Meyer KB, Martin AA, Fink NE, Coresh J, Powe NR, et al Comorbidity and its change predict survival in incident dialysis patients. Am J Kidney Dis. 2003;41: 149–161. 10.1053/ajkd.2003.50034 12500232

[11] Keith D, Ashby VB, Port FK, Leichtman AB. Insurance type and minority status associated with large disparities in prelisting dialysis among candidates for kidney transplantation. Clin J Am Soc Nephrol. 2008;3: 463–470. 10.2215/CJN.02220507 18199847 PMC2390941

[12] Sandhu GS, Khattak M, Pavlakis M, Woodward R, Hanto DW, Wasilewski MA, et al Recipient’s unemployment restricts access to renal transplantation. Clin Transplant. 2013;27: 598–606. 10.1111/ctr.12177 23808849

[13] Schaeffner ES, Mehta J, Winkelmayer WC. Educational level as a determinant of access to and outcomes after kidney transplantation in the United States. Am J Kidney Dis. 2008;51: 811–818. 10.1053/j.ajkd.2008.01.019 18436092

[14] Hommel K, Rasmussen S, Kamper A-L, Madsen M. Regional and social inequalities in chronic renal replacement therapy in Denmark. Nephrol Dial Transplant. 2010;25: 2624–2632. 10.1093/ndt/gfq110 20207710

[15] Young EW, Mauger EA, Jiang KH, Port FK, Wolfe RA. Socioeconomic status and end-stage renal disease in the United States. Kidney Int. 1994;45: 907–911. 8196296 10.1038/ki.1994.120

[16] Goldfarb-Rumyantzev AS, Sandhu GS, Baird BC, Khattak M, Barenbaum A, Hanto DW. Social Adaptability Index predicts access to kidney transplantation. Clin Transplant. 2011;25: 834–842. 10.1111/j.1399-0012.2010.01391.x 21269329

[17] Schold JD, Gregg JA, Harman JS, Hall AG, Patton PR, Meier-Kriesche H-U. Barriers to evaluation and wait listing for kidney transplantation. Clin J Am Soc Nephrol. 2011;6: 1760–1767. 10.2215/CJN.08620910 21597030

[18] Saunders MR, Cagney KA, Ross LF, Alexander GC. Neighborhood poverty, racial composition and renal transplant waitlist. Am J Transplant. 2010;10: 1912–1917. 10.1111/j.1600-6143.2010.03206.x 20659097

[19] Dudley CRK, Johnson RJ, Thomas HL, Ravanan R, Ansell D. Factors that influence access to the national renal transplant waiting list. Transplantation. 2009;88: 96–102. 10.1097/TP.0b013e3181aa901a 19584687

[20] Morton RL, Schlackow I, Mihaylova B, Staplin ND, Gray A, Cass A. The impact of social disadvantage in moderate-to-severe chronic kidney disease: an equity-focused systematic review. Nephrol Dial Transplant. 2015; 10.1093/ndt/gfu394 25564537

[21] Perneger TV, Brancati FL, Whelton PK, Klag MJ. End-stage renal disease attributable to diabetes mellitus. Ann Intern Med. 1994;121: 912–918. 7978716 10.7326/0003-4819-121-12-199412150-00002

[22] Kihal-Talantikite W, Deguen S, Padilla C, Siebert M, Couchoud C, Vigneau C, et al Spatial distribution of end-stage renal disease (ESRD) and social inequalities in mixed urban and rural areas: a study in the Bretagne administrative region of France. Clin Kidney J. 2015;8: 7–13. 10.1093/ckj/sfu131 25713704 PMC4310433

[23] Joshi S, Gaynor J, Ciancio G. Review of ethnic disparities in access to renal transplantation. Clin Transplant. 2012;26: E337–343. 10.1111/j.1399-0012.2012.01679.x 22775991

[24] Couchoud C, Stengel B, Landais P, Aldigier J-C, de Cornelissen F, Dabot C, et al The renal epidemiology and information network (REIN): a new registry for end-stage renal disease in France. Nephrol Dial Transplant. 2006;21: 411–418. 10.1093/ndt/gfi198 16234286

[25] Strang WN, Tuppin P, Atinault A, Jacquelinet C. The French organ transplant data system. Stud Health Technol Inform. 2005;116: 77–82. 16160239

[26] van Eupen M, Metzger MJ, Pérez-Soba M, Verburg PH, van Doorn A, Bunce RGH. A rural typology for strategic European policies. Land Use Policy. 2012;29: 473–482. 10.1016/j.landusepol.2011.07.007

[27] Organisation for Economic Co-operation and Development. Creating rural indicators for shaping territorial policy Paris; Washington, D.C.: Organisation for Economic Co-operation and Development; OECD Publications and Information Centre [distributor]; 1994.

[28] Vard T, Willems E, Lemmens E, Peters R. Use of the CORINE land cover to identify the rural character of communes and regions at EU level. Trends of some agri-environmental indicators of the European Union, EUR 21669. 2005.

[29] Lalloué B, Monnez J-M, Padilla C, Kihal W, Le Meur N, Zmirou-Navier D, et al A statistical procedure to create a neighborhood socioeconomic index for health inequalities analysis. Int J Equity Health. 2013;12: 21 10.1186/1475-9276-12-21 23537275 PMC3621558

[30] Garg J, Karim M, Tang H, Sandhu GS, DeSilva R, Rodrigue JR, et al Social adaptability index predicts kidney transplant outcome: a single-center retrospective analysis. Nephrol Dial Transplant. 2012;27: 1239–1245. 10.1093/ndt/gfr445 22036942

[31] Axelrod DA, Dzebisashvili N, Schnitzler MA, Salvalaggio PR, Segev DL, Gentry SE, et al The interplay of socioeconomic status, distance to center, and interdonor service area travel on kidney transplant access and outcomes. Clin J Am Soc Nephrol. 2010;5: 2276–2288. 10.2215/CJN.04940610 20798250 PMC2994090

[32] Wolfe RA, Ashby VB, Milford EL, Bloembergen WE, Agodoa LY, Held PJ, et al Differences in access to cadaveric renal transplantation in the United States. Am J Kidney Dis. 2000;36: 1025–1033. 10.1053/ajkd.2000.19106 11054361

[33] Alexander GC, Sehgal AR. Barriers to cadaveric renal transplantation among blacks, women, and the poor. JAMA. 1998;280: 1148–1152. 9777814 10.1001/jama.280.13.1148

[34] Sequist TD, Narva AS, Stiles SK, Karp SK, Cass A, Ayanian JZ. Access to renal transplantation among American Indians and Hispanics. Am J Kidney Dis. 2004;44: 344–352. 15264194 10.1053/j.ajkd.2004.04.039

[35] Satayathum S, Pisoni RL, McCullough KP, Merion RM, Wikström B, Levin N, et al Kidney transplantation and wait-listing rates from the international Dialysis Outcomes and Practice Patterns Study (DOPPS). Kidney Int. 2005;68: 330–337. 10.1111/j.1523-1755.2005.00412.x 15954924

[36] Garg PP, Frick KD, Diener-West M, Powe NR. Effect of the ownership of dialysis facilities on patients’ survival and referral for transplantation. N Engl J Med. 1999;341: 1653–1660. 10.1056/NEJM199911253412205 10572154

[37] Mathur AK, Ashby VB, Sands RL, Wolfe RA. Geographic variation in end-stage renal disease incidence and access to deceased donor kidney transplantation. Am J Transplant. 2010;10: 1069–1080. 10.1111/j.1600-6143.2010.03043.x 20420653

[38] Patzer RE, Amaral S, Wasse H, Volkova N, Kleinbaum D, McClellan WM. Neighborhood poverty and racial disparities in kidney transplant waitlisting. J Am Soc Nephrol. 2009;20: 1333–1340. 10.1681/ASN.2008030335 19339381 PMC2689891

[39] Roodnat JI, Laging M, Massey EK, Kho M, Kal-van Gestel JA, Ijzermans JNM, et al Accumulation of unfavorable clinical and socioeconomic factors precludes living donor kidney transplantation. Transplantation. 2012;93: 518–523. 10.1097/TP.0b013e318243030f 22298031

[40] Hall YN, O’Hare AM, Young BA, Boyko EJ, Chertow GM. Neighborhood poverty and kidney transplantation among US Asians and Pacific Islanders with end-stage renal disease. Am J Transplant. 2008;8: 2402–2409. 10.1111/j.1600-6143.2008.02413.x 18808403

[41] Kihal-Talantikite W, Padilla CM, Lalloué B, Gelormini M, Zmirou-Navier D, Deguen S. Green space, social inequalities and neonatal mortality in France. BMC Pregnancy Childbirth. 2013;13: 191 10.1186/1471-2393-13-191 24139283 PMC4015785

[42] Kihal-Talantikite W, Padilla CM, Lalloue B, Rougier C, Defrance J, Zmirou-Navier D, et al An exploratory spatial analysis to assess the relationship between deprivation, noise and infant mortality: an ecological study. Environ Health. 2013;12: 109 10.1186/1476-069X-12-109 24341620 PMC3882103

[43] Tonelli M, Klarenbach S, Rose C, Wiebe N, Gill J. Access to kidney transplantation among remote- and rural-dwelling patients with kidney failure in the United States. JAMA. 2009;301: 1681–1690. 10.1001/jama.2009.545 19383959

[44] O’Hare AM, Johansen KL, Rodriguez RA. Dialysis and kidney transplantation among patients living in rural areas of the United States. Kidney Int. 2006;69: 343–349. 10.1038/sj.ki.5000044 16408125

